# Relationship between sentinel lymph nodes and postoperative tangential fields in early breast cancer, evaluated using SPECT/CT

**DOI:** 10.1093/jrr/rrv035

**Published:** 2015-06-10

**Authors:** Koichi Wadasaki, Ikuno Nishibuchi

**Affiliations:** Department of Radiation Oncology, Hiroshima Prefectural Hospital, 1-5-54 Ujinakanda Minami-ku, Hiroshima 734-8530, Japan

**Keywords:** breast cancer, sentinel lymph node, SPECT/CT, whole-breast irradiation

## Abstract

Single-photon emission computed tomography/computed tomography (SPECT/CT) demonstrates the precise location of the sentinel lymph nodes (SLNs) in patients with breast cancer. We evaluated the relationship between SLNs and postoperative tangential fields by using SPECT/CT images. Subjects included 72 patients with early breast cancer who underwent SPECT/CT of the SLNs and received whole-breast irradiation with tangential fields after partial mastectomy. The SLN locations evaluated by using SPECT/CT images were entered into the treatment-planning CT image with a 5-mm-diameter sphere. A 15-mm-diameter sphere including the 5-mm treatment margin around the SLNs was defined as PTV-SLN. The PTV-SLN doses with tangential irradiation were evaluated and expressed as the percentage of the prescribed dose. In 69 patients, SLNs were detected by using SPECT/CT; 68 SLNs were located at axillary lymph node Level I, and one was located at Level II. A total of 62 SLNs (90%) were determined to be located inside the tangential fields on the digitally reconstructed radiography (DRR) images. The median doses of SLN center, mean PTV-SLN dose, and PTV-SLN D95 (the minimum dose delivered to 95% of the volume) were 94.1% (range, 15.3–101.9%), 93.7% (range, 29.3–104.0%) and 84.8% (range, 6.8–99.8%). The D95 for the SLNs with treatment margins were ≤90% of the prescribed doses in more than half of the cases. Modification of the individual treatment fields seemed to be necessary to ensure coverage of the SLNs in whole-breast irradiation.

## INTRODUCTION

The sentinel lymph nodes (SLNs) are those that first receive lymphatic drainages from the breast tissue. Accordingly, they are often the first and only sites of breast cancer metastases. SLN biopsy is the current standard procedure for early breast cancer without clinical lymph node metastases. One purpose of SLN biopsy is to determine the indication for axillary lymph node dissection [1]. However, two randomized studies showed that in certain groups of patients with early breast cancer, axillary lymph node dissection was not required, regardless of the SLN biopsy results [2, 3]. Another purpose of SLN biopsy is to guide systemic therapy decisions. The administration of systemic therapy is determined according to several factors regarding patient and tumor characteristics. Surveys about biological characteristics of tumor progressed and the impact of axillary lymph node status for the selection of systemic therapy are decreasing [4]. Accordingly, SLN biopsy itself may not be required in some situations for early breast cancer.

Post-operative radiation therapy after partial mastectomy contributes modestly to controlling occult axillary lymph node metastasis. This efficacy of radiation depends on the positional relationship of the occult lymph node metastasis and the radiation fields. There are some reports about the doses to axillary lymph node regions from postoperative whole-breast radiation, but the relationship between the SLN location and the whole-breast tangential field has not been sufficiently investigated.

Single-photon emission computed tomography/computed tomography (SPECT/CT) is a fusion of SPECT and CT that can demonstrate the precise SLN location prior to SLN biopsy [5]. Here we retrospectively reviewed the SPECT/CT images of the SLNs of patients with early breast cancer who received whole-breast irradiation after partial mastectomy and then evaluated the relationship between the SLNs and tangential radiation fields by entering the SLN locations into the treatment-planning CT images.

## MATERIALS AND METHODS

The subjects included 72 patients with early breast cancer who underwent SPECT/CT of the SLNs and whole-breast irradiation after partial mastectomy at our institution between April 2012 and March 2014. All patients were clinically node negative. Patients who received preoperative chemotherapy or hormone therapy, or regional lymph node irradiation were excluded.

Thirty-four patients underwent SPECT/CT the day before the operation, and 38 patients underwent SPECT/CT on the day of the operation. To the former patients 72 MBq ^99m^Tc-phytate was administered and to the latter patients 22 MBq ^99m^Tc-phytate was administered. Fifty-one patients were injected with the radioisotope (RI) periareolarly and 21 patients were injected both periareolarly and peritumorally. The injection site was manually massaged for 5 min, and SPECT/CT images were acquired 30 min after the injection by using a Symbia T16 (Siemens, Munich, Bayern, Germany). In acquiring SPECT/CT images, the posture of patients was almost same as for radiotherapy, with the arms raised up over the head.

**Fig. 1.**
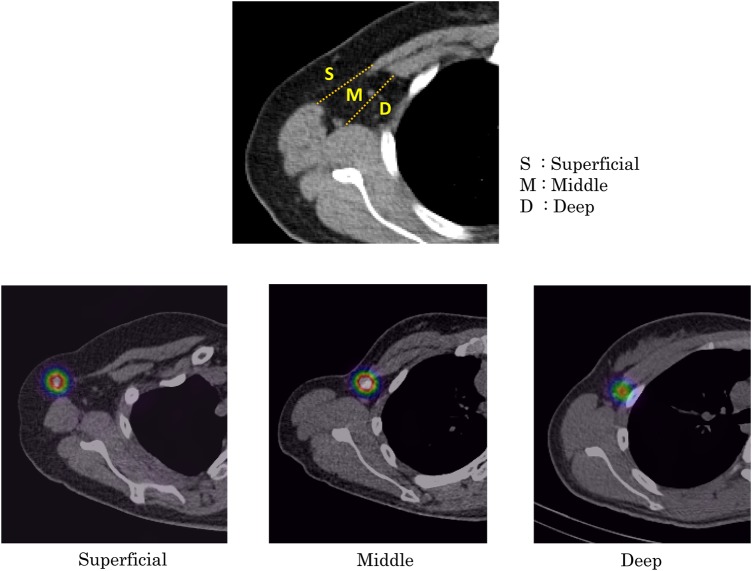
The axillary lymph node Level I is divided into three portions. ‘Superficial’ is anterior to the plane that connects the surface of the latissimus dorsi and pectoralis major muscles. The other portion is divided into the ‘middle’ and ‘deep’ sections considering the middle plane.

We considered the RI hot spots to be the SLNs. The SLN locations were classified as intrabreast, axillary Level I, Level II, Level III, supraclavicular and parasternal. Axillary Level I is divided into three subportions according to the depth. ‘Superficial’ is anterior to the plane that connects the surface of the latissimus dorsi and pectoralis major muscles. The other portion is divided into the ‘middle’ and ‘deep’ sections considering the middle plane (Fig. 1). The relationships between the rib level and SLN locations were also evaluated. In cases in which more than one SLN was detected, only those with the strongest RI accumulation were evaluated.

All patients underwent SLN biopsy and partial mastectomy. Two patients with macrometastasis in the SLN underwent axillary lymph node dissection. Postoperative whole-breast irradiation was delivered in tangential fields with 6-MV photons. CT-based 3D treatment-planning was performed by using the Pinnacle ver. 9.6 treatment-planning system (Philips Medical Systems, Fitchburg, WI, USA). The clinical target volume (CTV) was set to the whole breast, and the relationship between radiation fields and axillary lymph node regions was not considered. The breast borders were typically defined as follows: cranial = lower edge of the claviclar head, caudal = 1 cm below the inframammary fold, middle = center of the chest, lateral = middle axillary line; some modifications were applied by considering the doses to the lung or the heart. The point of dose evaluation for prescription was the center of the breast. Dose heterogeneity in the target was corrected by using the field-in-field technique to set the doses to as much of the CTV as possible at between 95 and 105% of the prescription dose. The prescribed dose was 50 Gy in 25 fractions for 32 patients and 42.6 Gy in 16 fractions for 40 patients. The median field length was 19.5 cm (range, 17.5–22.5 cm), and the median field width was 8.2 cm (range, 5.9–11.0 cm). The median beam angle of the medial fields from the horizontal plane was 33° (range, 20–48°). The median treatment depth was 6.0 cm (range, 3.0–10.2 cm).

**Fig. 2.**
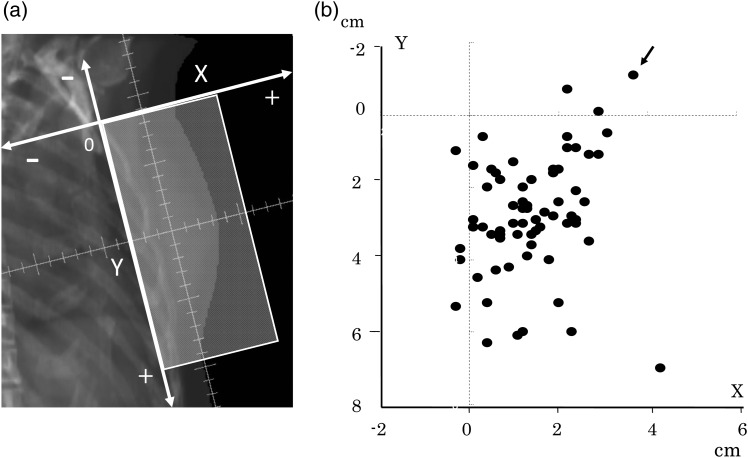
**(a)** Coordinates for evaluating the relationship between sentinel lymph nodes (SLNs) and lateral tangential field on a digitally reconstructed radiography image. The *x*-axis represents the anteroposterior direction, while the *y*-axis represents the craniocaudal direction. (**b)** The results of the distance from the SLN center to the field edge (inside the field was a positive value; outside the field was a negative value). The arrow indicates the case in whom the distance of the SLN center from the tangential field edge outward was the longest.

The SLN locations detected by using SPECT/CT were entered into the treatment-planning CT image, with a 5-mm-diameter sphere. The procedure was performed carefully with reference to the relationships between SLNs and the ribs or muscles because some postoperative changes existed in the surrounding tissues. The 15-mm-diameter sphere that included the 5-mm treatment margin around the SLNs was defined as PTV-SLN. The positional relationships between the SLNs and the fields were evaluated on digitally reconstructed radiography (DRR) images. The distances of the SLN center and field edges were measured (inside the field was a positive value, outside the field was a negative value; Fig. 2a). The PTV center dose, mean PTV-SLN dose and PTV-SLN D95 (the minimum dose delivered to 95% of the volume) were calculated from a dose distribution of the tangential whole-breast irradiation. These doses were expressed as percentages of the prescribed dose.

An analysis of the association of several factors with SLN doses from tangential fields was also performed. Analyzed factors were patient age, patient body mass index (BMI), tumor location, field length and width, field angle, treatment depth, SLN depth and SLN rib level. To test for significant differences, Spearman's correlation coefficient in the rank test was used for the continuous variables, and the Kruskal–Wallis test was used for the categorical variables. All statistical analyses were performed with Microsoft Excel 2010 (Microsoft, Redmond, WA, USA).

## RESULTS

### Patient characteristics

The median age of all 72 patients was 57 years (range, 26–83 years). The median BMI was 21.2 (range, 14.6–35.2). Clinical T-stage was Tis in 16 patients, T1 in 46 patients, and T2 in 10 patients. In 3 patients, the SLNs were not detected by using SPECT/CT. Consequently, the SLN locations and doses were evaluated for the other 69 patients. Table 1 shows the characteristics of all the patients as well as of the 69 patients in whom the SLNs were detected.

**Table 1.** Patient characteristics

Characteristics	All patients (*n* = 72)	Patients with SLN detected (*n* = 69)
Median age (range)	57 (26–83)	57 (26–79)
BMI	21.2 (14.6–35.2)	21.2 (14.6–35.2)
T stage		
Tis	16	16
T1	46	44
T2	10	9
Histology		
DCIS	17	17
IDC	44	41
ILC	4	4
Others	7	7
Hormone receptor		
Positive	62	59
Negative	10	10
HER2 status		
Positive	6	6
Negative	66	63
Tumor location		
Upper inner	16	15
Lower inner	9	9
Upper outer	35	33
Lower outer	10	10
Central	2	2

SLN = sentinel lymph node; BMI = body mass index; DCIS = ductal carcinoma *in situ*; IDC = invasive ductal carcinoma; ILC = invasive lobular carcinoma.

### SLN locations

The number of SLNs detected by using SPECT/CT was zero in 3 patients, one in 58 patients, two in 8 patients, three in 2 patients, and four in 1 patient. SLN locations in the 58 patients in whom one SLN was detected were all axillary lymph node Level I. In the 11 patients in whom more than one SLN was detected, the SLN with the strongest RI accumulation was axillary lymph node level I (10 patients) or axillary lymph node Level II (1 patient). Subclassified portions of these 69 SLNs were Level I superficial in 18, Level I middle in 40, Level I deep in 10, and Level II in 1 patient (Table 2). The relationships between the rib level and SLN location are shown in Table 2. Eighty-three percent (57/69) of the SLNs were between the second and third intercostal levels, with the second intercostal level being the most frequently affected.

**Table 2.** Locations of SLN

	Patient number
Level and depth	
Level I, superficial	18
Level I, middle	40
Level I, deep	10
Level II	1
Rib level	
2nd rib	9
2nd intercostal	34
3rd rib	13
3rd intercostal	10
4th rib	2
4th intercostal	1

### Relationships between SLN location and tangential fields

Sixty-two SLNs (90%) were located inside the lateral tangential fields according to the DRR images, three SLNs were located cranially outside the tangential fields, and four SLNs were located posteriorly outside the tangential fields (Fig. 2b). The median distance from the SLN center to the cranial field edge was 28 mm (range, −11 to 70 mm), while the median distance from the SLN center to the posterior field edge was 12 mm (range, −3 to 43 mm). In 32 patients (46%), the distance from the SLN center to the field edge was <1 cm. The longest distance of the SLN center from the tangential field edge outward was −11 mm (Fig. 2b).

### SLN doses

**Fig. 3.**
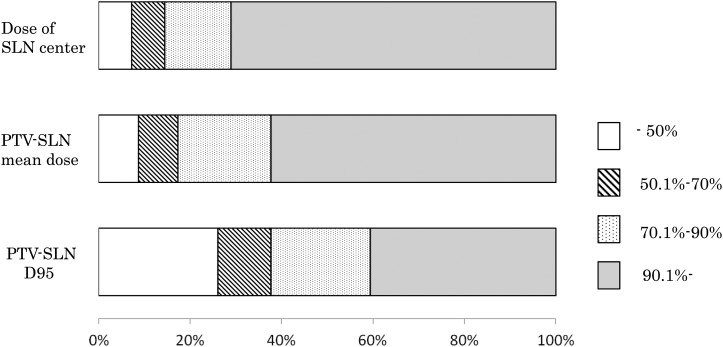
Distribution of patient numbers according to the doses to the SLN center, PTV-SLN mean doses, and PTV-SLN D95.

The median dose to the SLN center was 94.1% (range, 15.3–101.9%); it was >90% in 49 patients (71%). The median dose of the mean PTV-SLN dose was 93.7% (range, 29.3–104.0%), and 43 patients (62%) had values >90%. The median dose to the PTV-SLN D95 was 84.8% (range, 6.8–99.8%), and 28 patients (41%) had a value of >90%. Figure 3 shows the percentage of patients classified according to the dose range for the SLN center, the mean PTV-SLN dose, and D95 of the PTV-SLN.

Table 3 shows the association between several factors and the SLN dose on univariate analysis. The examined factors were age, BMI, tumor location, field length, field width, field angle, treatment depth, SLN depth, and SLN rib level. Of these factors, only the SLN depth was significantly correlated with the mean PTV-SLN dose and PTV-SLN D95. Evaluating the doses separately according to the SLN depth, the median dose of the mean PTV-SLN dose was 95.8%, superficial; 94.0%, middle; and 60.0%, deep. The median dose of the PTV-SLN D95 was 90.7%, superficial; 87.4%, middle; and 19.5%, deep.

**Table 3.** Univariate analysis for association of factors with SLN dose

Factors	PTV-SLN mean dose	PTV-SLN D95
	*P* value	*P* value
Age	0.2927	0.1351
BMI	0.2639	0.2529
Tumor location	0.3340	0.2966
Radiation field length	0.4955	0.3594
Radiation field width	0.9157	0.7128
Radiation field angle	0.5659	0.9304
Treatment depth	0.2567	0.2253
Depth of SLN	0.0013	0.0019
Rib level of SLN	0.9793	0.4374

## DISCUSSION

Several randomized studies have shown the non-inferiority of SLN biopsy alone compared with axillary lymph node dissection for SLN-negative patients with breast cancer [1, 6]. SLN biopsy is currently the standard procedure for patients with clinically node-negative breast cancer. Blue dye and/or RI are usually used to detect the SLNs. In the RI method, lymphoscintigraphy is performed to acquire preoperative SLN maps; at some institutions, SPECT/CT is also employed. In our institution, since April 2012, SPECT/CT has been performed before SLN biopsy in all patients. SPECT/CT can determine the precise anatomical locations of the SLNs and provide helpful information to surgeons [5, 7]. The SLNs most frequently exist in the axillary lymph node Level I region [8], but they can also be located in the Level II, Level III, or parasternal regions. In the present study, the SLNs of 68 of 69 patients were in the axillary lymph node Level I, and no SLNs were located in the parasternal region. We evaluated only the one SLN with the strongest RI accumulation if more than one SLN was detected. We used the periareolar injection site for the RI in most patients. These are probably the reasons that most SLNs were in axillary Level I in this study. Injected sites are reported to correlate with SLN location detection, and parasternal SLNs were detected more frequently when a peritumoral injection was used [9].

The recent ACOSOG Z0011 trial showed that, in cases of limited SLNs metastatic breast cancer, the use of SLN dissection alone did not result in inferior survival compared with axillary lymph node dissection [2]. In this trial, most patients underwent whole-breast irradiation after partial mastectomy, which was thought to contribute to some extent to the control of occult non-sentinel lymph node metastasis. The AMAROS study revealed that, in patients with positive SLNs, there was no difference in the treatment results between axillary lymph node dissection and axillary irradiation [10]. Accordingly, the relationship between whole-breast irradiation fields and axillary lymph node region becomes worthy of investigation. There are some reports about axillary lymph node dose in whole-breast tangential irradiation. Reznik *et al.* reported that the mean dose to the axillary Level I region from tangential fields designed to treat only the breast was 66% of the prescribed dose [11]. Reed *et al.* reported that the mean volume ratio of axillary Levels I–II that received ≥95% of prescribed dose was 55% and that no patient received complete coverage of the axillary Levels I–II lymph node volume [12]. They concluded that definitive irradiation of the Level I and II axillary lymph node region required significant modification of the standard tangential fields.

**Fig. 4.**
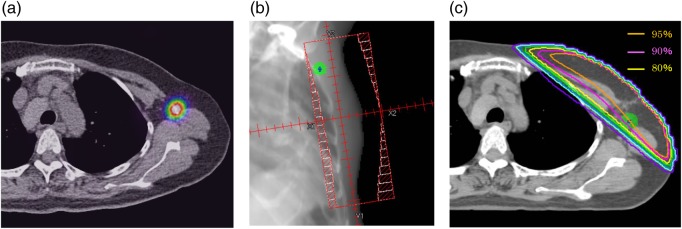
Patient example: (**a**) a SPECT/CT image, (**b**) a DRR image, (**c**) the dose distribution. Green circle = PTV-SLN. The SLNs are located in the middle portion of the axillary lymph node Level I. The PTV-SLN is located within the tangential field on DRR. The mean PTV-SLN dose is 92%, and the PTV-SLN D95 is 85%.

The SLNs are the most frequent (and sometimes only) lymph nodes affected by breast cancer metastases. In some cases of early breast cancer, an axillary lymph node procedure including SLN biopsy may not be required. As such, the relationship between the whole-breast irradiation field and the SLN location also seems worthy of investigation; however, studies evaluating SLN doses in whole-breast irradiation are scarce. Schlembach *et al.* reported on the relationship between SLN locations and tangential fields used in breast irradiation [13]. In their study, the median dose to the SLN region was 98% of the prescribed dose; in only 1 of 65 patients, the dose to the SLN region was <90% of the prescribed dose. They employed surgical clips as anatomic landmarks of the SLN region. In our study, the SLNs were located inside the tangential fields in 90% of patients, but the distance from the SLN center to the field edge was <1 cm in 46% of patients. The mean PTV-SLN doses that included treatment margins to the SLNs were ≤90% of prescribed doses in 38% of patients, and the D95 of the PTV-SLN was ≤90% of the prescribed doses in 59% of patients. Especially in patients in whom the SLNs were located within the deep portion of Level I, SLN coverage was poor. In some patients, the SLN doses were <90% of the prescribed doses according to the tangential thickness of the axillary region, despite being located within the tangential fields (Fig. 4). As described above, in our results, for many patients the SLNs were not covered adequately by the tangential fields. The major difference between the study by Schlembach *et al.* and ours is the method of determining the SLN locations; they used surgical clips and we used SPECT/CT. SPECT/CT depicts the SLN location by point, and the influence from postsurgical changes may be less than that with surgical clips. Several other factors are involved in the differences in results between these studies: racial differences in breast size and physique, radiation techniques, field extent, and technique for correcting dose heterogeneity probably influence the SLN doses. As such, the current results should be interpreted with caution.

Although many SLNs were not covered adequately by the tangential fields determined in this study, no SLNs existed far outside the field edges. The largest distance from the SLN center to the field edge outside was 11 mm. In situations in which it is preferable to include the SLNs in the radiation target, we think it is possible to intentionally increase SLN doses. The modified tangential irradiation technique (in which the field is adjusted wider than the traditional standard field in the cranial and posterior directions to ensure inclusion of the axillary regions) may be useful [14]. However, the SLN doses were lower than the prescribed doses in some patients due to the tangential thickness of the axillary area, despite them being within the tangential fields (Fig. 4). In these patients, to correct the axillary doses, the addition of small field boosts to the axillary area is a promising method, and the use of intensity-modulated radiation therapy may be helpful.

In conclusion, we evaluated the position of SLNs by using SPECT/CT images in patients with early breast cancer. The SLNs were located inside the tangential fields in >90% of cases. But in more than half of the cases, D95 of the SLNs with treatment margins was ≤90% of the prescribed doses. Modification of the individual treatment fields seemed to be necessary to ensure coverage of the SLNs in whole-breast irradiation.
